# Type IV Takayasu Arteritis Presenting as a Late-Onset Hypertensive Emergency and Transient Ischemia: A Case Report Highlighting Diagnostic Challenges

**DOI:** 10.7759/cureus.111141

**Published:** 2026-06-19

**Authors:** Amro Al-Zaben, Zaid El-Ebeisat, Ibrahim Al-Adayleh, Diala Al-Sukhon, Ahmed M Saleh

**Affiliations:** 1 Faculty of Medicine, AlBalqa Applied University, Salt, JOR; 2 Faculty of Medicine, Hashemite University, Zarqa, JOR; 3 Faculty of Medicine, University of Jordan, Amman, JOR; 4 Internal Medicine, School of Medicine, Wayne State University, Detroit, USA

**Keywords:** aortitis, hypertensive emergency, large vessel vasculitis, late-onset vasculitis, stenosis of the renal artery, takayasu arteritis (ta)

## Abstract

A 69-year-old female patient presented with a hypertensive emergency and recurrent episodes of left upper extremity paresthesia. While initial cerebrovascular imaging was negative, physical examination revealed a significant blood pressure discrepancy between the upper extremities. Subsequent computed tomography angiography of the abdomen identified abnormal circumferential wall thickening of the infrarenal abdominal aorta extending into the common iliac arteries, consistent with aortitis. Additionally, duplicate left renal arteries were noted, with severe stenosis of the posterior artery driving the hypertensive crisis. These findings supported a diagnosis of Type IV Takayasu arteritis, an exceptional presentation in a patient of advanced age with normal inflammatory markers. The patient was stabilized with intravenous antihypertensive therapy and transferred for multidisciplinary management. This case underscores the importance of considering large-vessel vasculitis in the differential diagnosis of elderly patients presenting with unexplained hypertensive emergencies, as reliance on age demographics or inflammatory markers may delay accurate diagnosis.

## Introduction

Takayasu arteritis (TA), also known as "pulseless disease," is a systemic inflammatory condition characterized by granulomatous vasculitis that primarily affects the aorta and its major branches. The disease predominantly affects women of Asian descent and typically presents in the second or third decade of life [1]. The incidence of TA is estimated at 1-2.6 cases per million annually. Common complications include heart failure, stroke, and irreversible end-organ damage, while prognosis largely depends on timely immunosuppression [1]. Aortitis, the general term for inflammation of the aorta, is mostly caused by large-vessel vasculitides such as TA and giant cell arteritis (GCA) [2]. While TA is classically defined by an age of onset under 40 years, cases of late-onset disease in the elderly have been described. However, they are rare and often present with atypical features that mimic atherosclerosis [3].

Renal artery stenosis (RAS) is a critical complication of TA, resulting from transmural fibrous thickening that leads to narrowing of the vessel lumen [1]. It is a major cause of secondary hypertension, mediated by the activation of the renin-angiotensin-aldosterone system (RAAS) due to renal ischemia [4]. We present a case of a 69-year-old female patient with no significant past medical history who presented with a hypertensive emergency and transient upper extremity ischemia. Late-onset disease can radiologically and clinically mimic atherosclerotic disease due to the shared presentation of vessel narrowing and reduced pulses, posing a significant diagnostic challenge in atypical presentations [1].

## Case presentation

A 69-year-old female real estate professional presented to the emergency department with a chief complaint of sudden onset of numbness in the left arm and chin. The numbness extended from the left elbow down to the digits and was associated with a sensation of discomfort. The patient reported that the numbness in her chin resolved within seconds, but the sensation in her left arm persisted for approximately four hours. She noted a history of similar episodes over the past year, always affecting the same distribution in the left arm, typically lasting about one minute and resolving with limb shaking.

Upon arrival, the patient was found to be in a hypertensive emergency. Blood pressure in the left arm was recorded as 209/96 mmHg, while the right arm was 120 mmHg by palpation, indicating a significant discrepancy between the upper extremities. Her respiratory rate was 18 breaths/minute, heart rate was 88 beats/minute, and oxygen saturation was 98% on room air. Neurological examination upon arrival was unremarkable, with a Glasgow Coma Scale score of 15 (E4V5M6), cranial nerves II-XII intact, normal muscle strength (5/5) in all extremities, intact sensation to light touch, and no cerebellar signs. The National Institutes of Health Stroke Scale score was zero [5]. The patient denied chest pain, shortness of breath, palpitations, nausea, or vomiting. She reported no history of tobacco or alcohol use. Her family history was significant for a massive myocardial infarction in her father at age 60 and Alzheimer's disease in her mother.

She was administered 5 mg of hydralazine intravenously. The clinical rationale in this hypertensive emergency was to achieve a controlled, gradual reduction of blood pressure rather than immediate normalization to prevent cerebral ischemia and organ hypoperfusion. Following administration, her blood pressure decreased to 158/62 mmHg, and continuous heart rate monitoring confirmed a stable sinus rhythm without reflex tachycardia.

On physical examination, the patient was awake, alert, and in no acute distress. The head and neck exam was atraumatic and normocephalic. Cardiac auscultation revealed a regular rate and rhythm with no murmurs, gallops, or rubs. Lungs were clear to auscultation bilaterally. The abdominal exam was soft and nontender, with no bruits noted. Extremities showed no clubbing, cyanosis, or edema. Radial pulses were palpable and symmetric (2+) despite the blood pressure discrepancy noted on the automated cuff.

Initial laboratory investigations were largely unremarkable. A complete blood count and comprehensive metabolic panel were within normal limits, including renal function. Notably, inflammatory markers were not elevated, with a C-reactive protein (CRP) of 1.90 mg/L. A summary of the initial laboratory results is provided in Table 1.

**Table 1:** Initial laboratory results Initial laboratory values were obtained at the time of presentation, including renal, hepatic, and inflammatory parameters AST: aspartate aminotransferase; ALT: alanine aminotransferase

Parameters	Result	Unit	Reference range
Sodium	141	mmol/L	135-145
Potassium	3.5	mmol/L	3.5-5.0
Chloride	107	mmol/L	98-107
Blood urea nitrogen	16	mg/dL	7-20
Creatinine	0.85	mg/dL	0.6-1.2
Glucose	89	mg/dL	70-100
Calcium	9.6	mg/dL	8.5-10.5
Total bilirubin	1	mg/dL	0.1-1.2
Alkaline phosphatase	99	U/L	44-147
AST	26	U/L	10-40
ALT	23	U/L	7-56
Total protein	7	g/dL	6.0-8.3
Albumin	3.9	g/dL	3.5-5.0
C-reactive protein	1.9	mg/L	< 5.0
Hemoglobin A1c	5	%	< 5.7
White blood cell count	8.6	×10^3^/µL	4.0-11.0
Hemoglobin	14.7	g/dL	12.0-16.0
Platelets	306	×10^3^/µL	150-450

A computed tomography angiography (CTA) of the chest was performed, identifying an aneurysmal change of the ascending thoracic aorta measuring 4 cm in diameter; however, there was no evidence of dissection, rupture, or abnormal aortic wall thickening in this segment, and the major branches of the aortic arch appeared widely patent (Figure 1).

**Figure 1:**
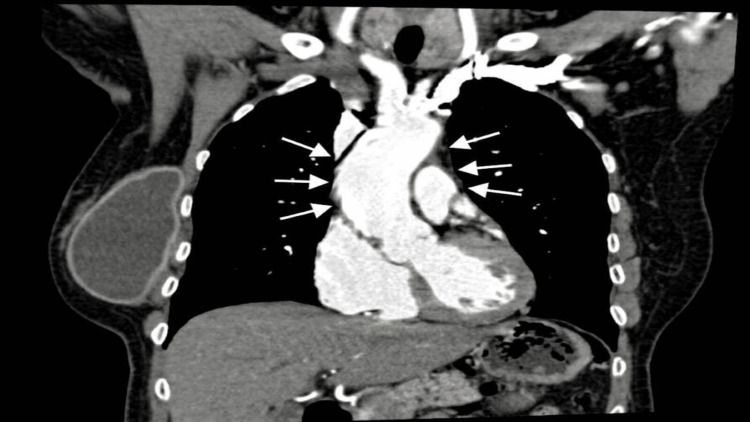
Computed tomography angiography of the chest Axial view revealing aneurysmal dilation of the ascending thoracic aorta measuring 4 cm in diameter, with no evidence of dissection or abnormal wall thickening

Given the persistent hypertension and clinical suspicion for renovascular pathology, a renal artery duplex ultrasound was performed, which showed elevated peak systolic velocities at the ostium of the left renal artery, suggestive of stenosis. To further evaluate this finding, a CTA of the abdomen and pelvis was obtained. The imaging revealed abnormal, circumferential wall thickening of the infrarenal abdominal aorta, measuring up to 4 mm thick (Figure 2).

**Figure 2:**
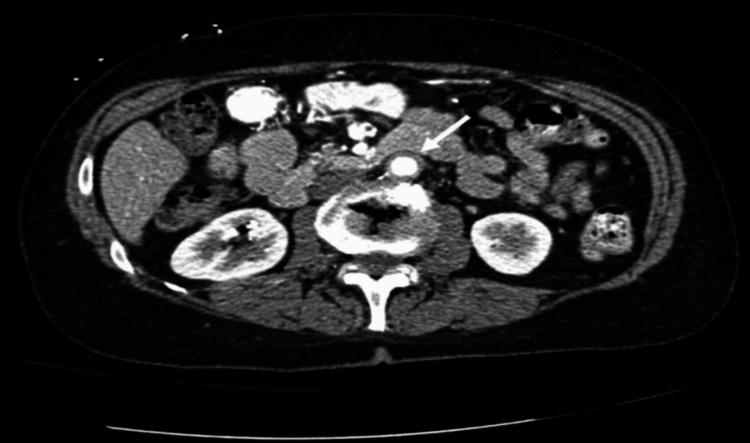
Computed tomography angiography of the abdomen and pelvis Coronal view demonstrating abnormal, circumferential wall thickening (up to 4 mm) of the infrarenal abdominal aorta, consistent with aortitis

This finding was radiologically consistent with aortitis, potentially representing early Type IV TA. Additionally, the scan revealed a duplication of the left renal arteries. The anterior left renal artery showed 30% stenosis, while the posterior left renal artery exhibited severe stenosis of approximately 75% (Figure 3). The right renal artery was widely patent. The radiologist noted an overall paucity of atherosclerotic calcifications, further supporting a vasculitic etiology over degenerative disease.

**Figure 3:**
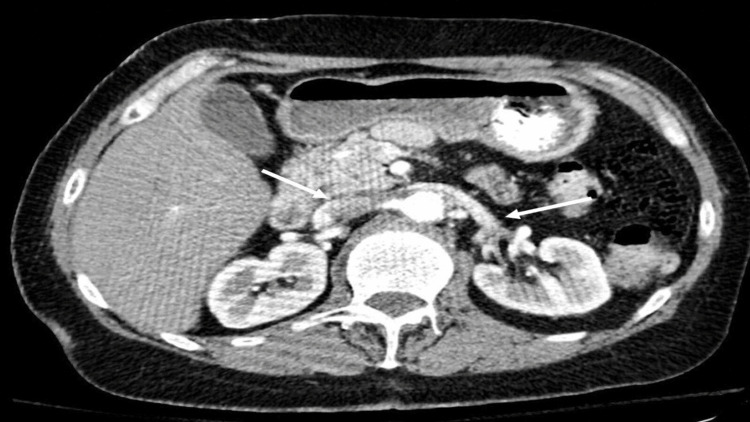
Computed tomography angiography of the renal arteries Axial view showing duplicate left renal arteries with severe (75%) stenosis of the posterior left renal artery and mild (30%) stenosis of the anterior left renal artery

The patient was admitted to the intensive care unit for close blood pressure monitoring and management with a nicardipine infusion. Her blood pressure stabilized, and she was transitioned to oral amlodipine and losartan (50 mg daily). The use of an angiotensin receptor blocker was clinically justified given the strictly unilateral nature of her RAS. Vascular surgery was consulted and recommended transfer to a tertiary care center for Rheumatology evaluation and management of suspected large-vessel vasculitis. The patient remained clinically stable, with no recurrence of numbness, for the remainder of her hospital stay and was transferred for specialized care. A timeline of the patient's clinical course is shown in Table 2.

**Table 2:** Timeline of the clinical course A summary of the patient's hospitalization, diagnostic workup, and management ED: emergency department; BP: blood pressure; NIHSS: National Institutes of Health Stroke Scale; CT: computed tomography; CTA: computed tomography angiography; ICU: intensive care unit; BP: blood pressure; IV: intravenous

Day	Clinical events
Day 1	Presented to ED with left arm and chin numbness
	BP discrepancy noted (left 209/96 mmHg vs. right 120/P)
	NIHSS 0; symptoms transient
	CT head and CTA brain/neck negative for acute stroke
	Admitted for hypertensive emergency; started on aspirin and clopidogrel
Day 2	Renal artery duplex ultrasound showed elevated velocities in left renal artery
Day 3	Recurrence of left arm numbness prompting rapid response; BP spike to 180s
	Transferred to ICU for nicardipine drip
	CT angiography abdomen performed: showed infrarenal aortic wall thickening (3-4 mm) and severe stenosis of the posterior left renal artery
	Vascular surgery consulted; diagnosis of aortitis (type IV Takayasu) suspected
	Patient stabilized on oral antihypertensives (amlodipine, losartan)
	Transfer initiated to the tertiary care center for rheumatology management

## Discussion

TA has historically been categorized as a "disease of the young," with the 1990 American College of Rheumatology (ACR) criteria requiring an age of onset under 40 years for classification [6]. Late-onset TA (LOTA), defined as disease onset after age 40, is an increasingly recognized entity. While classically associated with young women, approximately 15%-20% of patients may present later in life [3].

The diagnosis of aortitis in an elderly patient requires a careful exclusion of more common etiologies, primarily atherosclerosis and GCA [2]. Atherosclerosis is the most prevalent cause of aortic wall thickening in patients over 60 years; however, it typically presents with irregular, calcified plaques and is strongly associated with cardiovascular risk factors such as smoking and dyslipidemia [7]. Our patient’s pathology was strictly confined to the abdominal aorta and renal arteries, a distribution pattern more characteristic of TA. Angiographically, this specific pattern corresponds to Type IV vascular involvement under the schema formalized at the 1996 International Tokyo Conference (Hata classification) [8]. Furthermore, under the clinical complication grading system originally pioneered by Ishikawa in 1988 [9], our patient is classified as Group IIb, as she presented with exactly one severe major complication (a hypertensive emergency) without concurrent evidence of acute stroke or heart failure; these classifications are demonstrated in Table 3.

**Table 3:** Angiographic and clinical classification systems for Takayasu arteritis A comprehensive summary of the Hata/Tokyo Angiographic Classification System (1996) and the Ishikawa Clinical Severity Framework (1988) used to categorize anatomical distribution and disease severity in patients with Takayasu arteritis

System	Type/group	Anatomical distribution and clinical criteria
Hata/Tokyo Angiographic System (1996)	Type I	Brachiocephalic trunk, common carotid, and subclavian arteries
	Type IIa	Ascending aorta, aortic arch, and its branches
	Type IIb	Ascending aorta, aortic arch, its branches, and thoracic descending aorta
	Type III	Thoracic descending aorta, abdominal aorta, and/or renal arteries
	Type IV	Abdominal aorta and/or renal arteries strictly
	Type V	Panoramic involvement (combined features of Type IIb and Type III)
Ishikawa Clinical Severity Framework (1988)	Group I	Uncomplicated disease, with or without pulmonary artery involvement
	Group IIa	Exactly one mild or moderate major complication present
	Group IIb	Exactly one severe, major complication present
	Group III	Two or more severe, major complications present simultaneously

Furthermore, our patient was a nonsmoker with a paucity of calcified plaques on computed tomography imaging, and the aortic thickening was circumferential and homogeneous, findings radiologically distinctive for vasculitis rather than degenerative disease [10]. GCA is another major consideration in this age group but predominantly affects the thoracic aorta and cranial branches [2]. Additionally, the absence of systemic infectious signs and the chronic, recurrent nature of her symptoms argued against infectious aortitis.

A limitation of our clinical assessment during the initial presentation was the absence of blood pressure measurements across all four extremities. It is strongly advised to measure arterial tension in both upper and lower limbs to fully evaluate hemodynamic discrepancies in suspected large-vessel vasculopathy.

The clinical presentation of TA in the elderly can be highly atypical. In patients over 60 years, distinguishing TA from GCA is particularly difficult; however, LOTA patients tend to exhibit more extensive involvement of the abdominal aorta and renal arteries compared to the thoracic predominance seen in GCA [11]. A recent case report by Gouveia et al. described an 87-year-old female patient diagnosed with TA who presented with neurological manifestations (syncope and amaurosis fugax) rather than classic constitutional symptoms [12]. Similarly, our 69-year-old patient presented with neurological symptoms (recurrent limb and chin paresthesia) secondary to hypertensive urgency. These cases underscore that TA is not exclusively a disease of the young and should remain on the differential for unexplained large-vessel vasculopathy in older adults, even those in their eighth or ninth decade of life.

While the 1990 ACR criteria heavily weighed clinical findings such as pulse deficits and bruits, modern diagnostic algorithms increasingly prioritize cross-sectional imaging [6]. The 2022 ACR/European League Against Rheumatism classification criteria have formally incorporated advanced imaging, recognizing that CTA and magnetic resonance angiography are now standard for diagnosis [13]. Key imaging features include circumferential wall thickening (often greater than 2-3 mm), luminal stenosis, and mural enhancement indicative of active inflammation [14]. In this patient, CTA demonstrated circumferential wall thickening of the infrarenal aorta extending into the common iliac arteries. This finding is analogous to the "macaroni sign" typically seen in ultrasound imaging of TA, which reflects marked, concentric thickening of the intimal-medial complex [14].

The patient's hypertensive emergency was likely driven by the critical stenosis of the posterior left renal artery. In RAS, the underlying arteritis causes critical anatomical narrowing, which induces chronic renal ischemia. This ischemia triggers the release of renin from the juxtaglomerular apparatus, activating the RAAS. It is this RAAS excitation, not the arteritis directly, that causes potent vasoconstriction and aldosterone-mediated sodium and water retention, driving the secondary hypertensive emergency [4]. A confounding factor in this case was the normal level of CRP. While elevated inflammatory markers are often seen in active vasculitis, they are unreliable for monitoring disease activity in TA [15]. Up to 23% of patients with histologically active disease may present with normal acute phase reactants, a state often referred to as "burnt-out" or quiescent disease [10]. Consequently, the absence of elevated inflammatory markers in our patient did not exclude the diagnosis, particularly given the strong imaging evidence of vessel wall pathology. Management typically begins with corticosteroids, although relapse rates are high, and long-term immunosuppression or revascularization is often required to prevent irreversible end-organ damage [10].

The prognosis of TA in the elderly population presents unique challenges. Advanced age and superimposed cardiovascular risk factors often contribute to higher morbidity and poorer outcomes compared to younger cohorts. Relapse rates remain significant, requiring careful long-term multidisciplinary management to mitigate ischemic complications [16,17].

## Conclusions

This case serves as a critical reminder that TA should not be dismissed solely based on advanced patient age or the absence of systemic inflammation. The presentation of a hypertensive emergency in a geriatric patient, while statistically more likely to be atherosclerotic, may mask an underlying large-vessel vasculitis. The striking disparity between the profound anatomical involvement, characterized by the pathognomonic "macaroni sign," and the quiescent serological profile underscores the limitations of traditional acute-phase reactants in this demographic. Ultimately, a high index of clinical suspicion and a reliance on advanced cross-sectional imaging are paramount. This case advocates a paradigm shift in the diagnostic approach to renovascular hypertension, suggesting that systemic vasculitis must remain a differential consideration regardless of the patient's age or the perceived inactivity of biological markers.

## References

[1] Trinidad B, Surmachevska N, Lala V (2023). Takayasu Arteritis. https://www.ncbi.nlm.nih.gov/books/NBK459127/.

[2] Gornik HL, Creager MA (2008). Aortitis. Circulation.

[3] Tombetti E, Sarzi-Puttini P (2026). Takayasu arteritis: recent advances and clinical pitfalls - beyond rheumatology. Beyond Rheumatol.

[4] Bokhari MR, Bokhari SRA (2023). Renal Artery Stenosis. https://www.ncbi.nlm.nih.gov/books/NBK430718/.

[5] Brott T, Adams HP Jr, Olinger CP (1989). Measurements of acute cerebral infarction: a clinical examination scale. Stroke.

[6] Arend WP, Michel BA, Bloch DA (1990). The American College of Rheumatology 1990 criteria for the classification of Takayasu arteritis. Arthritis Rheum.

[7] Jebari-Benslaiman S, Galicia-García U, Larrea-Sebal A (2022). Pathophysiology of atherosclerosis. Int J Mol Sci.

[8] Hata A, Noda M, Moriwaki R, Numano F (1996). Angiographic findings of Takayasu arteritis: new classification. Int J Cardiol.

[9] Ishikawa K (1988). Diagnostic approach and proposed criteria for the clinical diagnosis of Takayasu's arteriopathy. J Am Coll Cardiol.

[10] Kerr GS, Hallahan CW, Giordano J, Leavitt RY, Fauci AS, Rottem M, Hoffman GS (1994). Takayasu arteritis. Ann Intern Med.

[11] Gribbons KB, Ponte C, Carette S (2020). Patterns of arterial disease in Takayasu arteritis and giant cell arteritis. Arthritis Care Res (Hoboken).

[12] Gouveia F, Ferreira S, Lemos C (2024). Takayasu arteritis - a case report of neurological clinical manifestation in an elderly patient. J Neurol Stroke.

[13] Grayson PC, Ponte C, Suppiah R (2022). 2022 American College of Rheumatology/EULAR classification criteria for Takayasu arteritis. Ann Rheum Dis.

[14] Ghembaza ME, Boulenouar F, Lounici A (2018). "Macaroni sign" in Takayasu arteritis. J Cardiovasc Imaging.

[15] O'Connor TE, Carpenter HE, Bidari S, Waters MF, Hedna VS (2014). Role of inflammatory markers in Takayasu arteritis disease monitoring. BMC Neurol.

[16] Cozzi F, Botsios C, Ostuni P (2002). Adult Henoch-Schönlein purpura with glomerulonephritis and paroxysmal nocturnal haemoglobinuria: an uncommon association. Clin Rheumatol.

[17] Soto ME, Espinola N, Flores-Suarez LF, Reyes PA (2008). Takayasu arteritis: clinical features in 110 Mexican Mestizo patients and cardiovascular impact on survival and prognosis. Clin Exp Rheumatol.

